# Brain Structural Correlates of Dispositional Insight and the Mediation Role of Neuroticism in Young Adults

**DOI:** 10.3389/fnbeh.2022.846377

**Published:** 2022-04-15

**Authors:** Jiabao Lin, Yajue Chen, Jiushu Xie, Qiuping Cheng, Mi Zou, Lei Mo

**Affiliations:** ^1^Department of Psychology, School of Public Health and Management, Guangzhou University of Chinese Medicine, Guangzhou, China; ^2^Guangdong Key Laboratory of Mental Health and Cognitive Science, Center for Studies of Psychological Application, School of Psychology, South China Normal University, Guangzhou, China; ^3^Key Laboratory of Brain, Cognition and Education Sciences, South China Normal University, Ministry of Education, Guangzhou, China; ^4^School of Civil and Transportation Engineering, Guangdong University of Technology, Guangzhou, China; ^5^School of Psychology, Nanjing Normal University, Nanjing, China

**Keywords:** dispositional insight, structural brain mechanism, voxel-based morphometry, functional connectivity, mediation analysis

## Abstract

Studies on the neural correlates of episodic insight have made significant progress in the past decades. However, the neural mechanisms underlying dispositional insight are largely unknown. In the present study, we recruited forty-four young, healthy adults and performed several analyses to reveal the neural mechanisms of dispositional insight. Firstly, a voxel-based morphometry (VBM) technique was used to explore the structural brain mechanisms of dispositional insight. We found that dispositional insight was significantly and negatively correlated with the regional gray matter volume (rGMV) in the left thalamus (TLM.L), right temporoparietal junction (TPJ.R), and left dorsal medial prefrontal cortex (DMPFC.L). Secondly, we performed a seed-based resting-state functional connectivity (RSFC) analysis to complement the findings of VBM analysis further. The brain regions of TLM.L, DMPFC.L, and TPJ.R were selected as seed regions. We found that dispositional insight was associated with altered RSFC between the DMPFC.L and bilateral TPJ, between the TPJ.R and left dorsolateral prefrontal cortex, left ventrolateral prefrontal cortex, DMPFC.L, TPJ.L, right insula, and right cerebellum. Finally, a mediation analysis found that the personality of neuroticism partially mediated the relationship between the brain region of TLM.L and dispositional insight. These findings imply that dispositional insight has a specific functional and structural neural mechanism. The personality of neuroticism may play a pivotal role in the processes of dispositional insight.

## Introduction

Creativity is the primary human function to produce work and thoughts that are both original and valuable (Sternberg and Lubart, 1996; Beaty et al., 2018; Ren et al., 2020; Tang et al., 2022). The importance of creativity has long been demonstrated (Ren et al., 2020). Insight, a form of creativity, is a sudden comprehension that reinterprets a situation and solves a problem (Bowden and Jung-Beeman, 2003; Webb et al., 2016; Tik et al., 2018; Lin et al., 2020). Studies of insight properties usually focus on two dimensions: cognitive process and individual characteristics (Ash and Wiley, 2006; Ovington et al., 2016; Ogawa et al., 2018). Previous studies, treating insight as a problem’s solution process, used specialized tasks to induce insight and reveal the cognitive mechanisms under insightful solutions (Metcalfe and Wiebe, 1987; Knoblich et al., 1999; Kounios and Beeman, 2009). Overall, these studies measured episodic (state) insight. Alternatively, other studies treated insight as an individual characteristic and found reliable individual differences in solving insight problems (Jacobs and Dominowski, 1981; Ash and Wiley, 2006; Gilhooly and Fioratou, 2009; DeCaro and Wieth, 2016). For instance, a study found stable individual differences in performance across different object-use insight problems (Jacobs and Dominowski, 1981). Briefly, these studies measured dispositional (trait) insight. Studying dispositional insight can explain why some people have more insightful problem-solving success than others. Recently, many studies have utilized various paradigms, such as riddles solving, to investigate the neural mechanisms of episodic insight (Jung-Beeman et al., 2004; Zhao et al., 2014; Tik et al., 2018; Lin et al., 2020). However, the neural mechanisms of dispositional insight are rarely understood. In the current study, we utilized voxel-based morphometry (VBM) method to explore the structural neural mechanisms of dispositional insight.

Psychologically, episodic insight contains mental processes of set-shifting, reorienting attention, method-searching, emotional experience, and establishing new representations (Jung-Beeman et al., 2004; Qiu et al., 2010; Tik et al., 2018; Lin et al., 2020). For instance, when people came to an impasse in a complex problem, they needed to reconsider basic assumptions of the problem, reorient attention, and find a new method (Qiu et al., 2010; Tang et al., 2015). Moreover, insightful problem-solving can induce positive feelings (Jung-Beeman et al., 2004). Previous studies have shown that episodic and dispositional insight may share some common characteristics (Byrne and Murray, 2005; Ovington et al., 2016). For instance, similar to the findings of episodic insight, individuals with high dispositional insight had great attention switching capacity (Byrne and Murray, 2005) and positive affect (Ovington et al., 2016). In addition, compared to episodic insight, dispositional insight, a trait of personality, was found to be associated with high cognitive flexibility and tendency to engage in effortful mental activities, which may recruit more set-shifting and motivational components (Gilhooly and Murphy, 2005; Ovington et al., 2016).

Neurally, several underlying neural mechanisms in episodic insight have been described. Neuroimaging studies using functional magnetic resonance imaging (fMRI) indicated increased medial prefrontal cortex (MPFC), temporal, and parietal activity in episodic insight (Jung-Beeman et al., 2004; Kounios et al., 2006; Huang et al., 2018). For example, researchers employed a compound remote associates paradigm and found neural activity in the MPFC, superior temporal gyrus (STG), and anterior cingulate cortex (ACC) accompanied insightful problem solving (Jung-Beeman et al., 2004; Kounios et al., 2006; Tik et al., 2018). The occurrence of an insight experience was related to brain activation in the temporoparietal junction (TPJ) by using riddles as experimental materials (Huang et al., 2018). Together, these findings suggest that MPFC and TPJ may be vital for episodic insight. Considering the commonalities between episodic and dispositional insight and their uniqueness, we hypothesized that dispositional insight can involve particular neural mechanisms in addition to the common neural mechanisms engaged in episodic insight.

Recently, many studies used resting-state fMRI to explore the functional connectivity in creativity (Takeuchi et al., 2012; Beaty et al., 2014; Chen et al., 2014; Sun et al., 2018). For example, researchers detected that creative performance was associated with the resting-state functional connectivity (RSFC) between the inferior frontal gyrus and the default mode network (Beaty et al., 2014). Takeuchi et al. (2012) reported that creativity was related to RSFC between the MPFC and posterior cingulate cortex using the task of divergent thinking. Furthermore, a previous study has combined the VBM with RSFC to evaluate the brain structural-functional relationships in creativity. Researchers employed the creative achievement questionnaire (CAQ) to calculate individual creativity in this study. Results showed that creativity was negatively correlated with the dorsal ACC’s regional gray matter volume (rGMV). Creativity was also negatively associated with the RSFC between the dorsal ACC and medial superior frontal gyrus (MSFG) (Chen et al., 2014). However, few studies have used structural and functional analyses to explore the changed brain structure and RSFC in dispositional insight.

Over the past decades, many studies have explored the relationship between personality characteristics and creativity (King et al., 1996; Batey, 2007; Furnham and Bachtiar, 2008; Li et al., 2014; Sadana et al., 2021). For example, researchers used the NEO Personality Inventory and divergent thinking tests to study the linkage between personality and creativity. They found that creativity was significantly correlated with extraversion and openness to experience (King et al., 1996). Furthermore, several studies also identified that creativity potential was associated with high neuroticism (Batey, 2007; Furnham and Bachtiar, 2008). The findings mentioned above suggest that essential aspects of personality may play a pivotal role in creativity. In addition, researchers found that character was closely associated with brain structure (DeYoung et al., 2010; Kong et al., 2015b). For example, neuroticism was related to structural variations in the STG and thalamus (Kong et al., 2015b). Furthermore, a previous study has indicated that personality can mediate the relationship between brain structure and trait creativity (Li et al., 2014). Thus, we deduce that individual personality differences might mediate the relationship between dispositional insight and rGMV in particular brain regions.

Previous studies have validated that using individuals’ performance on insight problems can estimate dispositional insight (Jacobs and Dominowski, 1981; Ash and Wiley, 2006; DeCaro and Wieth, 2016; Ogawa et al., 2018). For instance, Jacobs and Dominowski (1981) used average performance (i.e., mean solution times) on several classic insight problems, such as candle problems and two-string problems, to calculate the dispositional insight of each subject. Results showed significant differences in insightful problem-solving performance between males and females. Recently, researchers utilized problem-solving success (i.e., mean correct solution rates and solution times) on insightful chunk decomposition problems (i.e., matchstick arithmetic problems) to calculate the dispositional insight of each subject. They found that working memory can predict an individual’s ability to successfully solve insightful problems (Ash and Wiley, 2006; DeCaro and Wieth, 2016). Notably, insightful chunk decomposition problems were typically regarded as insight problems (Knoblich et al., 1999; DeCaro and Wieth, 2016). For instance, in the matchstick arithmetic task (i.e., chunk decomposition task), subjects were required to transform wrong arithmetic equations, such as “XI = III+III,” into true equations, such as “VI = III + III.” To solve the problems, subjects should decompose the “X” into “V.” This process involved cognitive restructuring, the defining characteristic of insight problems (Knoblich et al., 1999). However, the number of matchstick arithmetic problems is minimal. Researchers have recently developed a new insightful chunk decomposition task (i.e., insightful Chinese character chunk decomposition task), which can provide a sufficient number of insight problems (Luo et al., 2006; Wu et al., 2009; Huang et al., 2015). Descriptions of the task can be found elsewhere (Luo et al., 2006; Wu et al., 2009; Huang et al., 2015). Thus, in the present study, we used individuals’ performance (i.e., mean correct solution rates and solution times) on insightful Chinese character chunk decomposition tasks to measure dispositional insight.

Together, we aimed to study the relevance between altered brain structure associated with RSFC and dispositional insight. Notably, the RSFC analysis was to complement the findings of VBM analysis in this study. We used multi-modal brain structural MRI and resting-state fMRI analyses to detect the structural and functional neural mechanisms underlying dispositional insight. Based on the studies mentioned above, we hypothesized that rGMV and RSFC in the MPFC and TPJ might correlate with dispositional insight. Furthermore, we predicted that subjects’ personalities might mediate the linkage between dispositional insight and rGMV in particular brain regions.

## Materials and Methods

The current study was conducted according to the Declaration of Helsinki. The Research Ethics Review Board of South China Normal University approved the present study. Subjects provided written informed consent before their participation in the current study.

### Subjects

The sample included forty-four subjects (18 males, mean age 20.73, range 18–27). We determined the sample size according to a previous neuroimaging study (Xiang et al., 2016). All the subjects were students from South China Normal University. They were screened by questionnaire to exclude subjects who have the history of neurological or psychiatric illness. All of them reported right-handed and had a normal or corrected-to-normal vision.

### Insight Task

**FIGURE 1:**
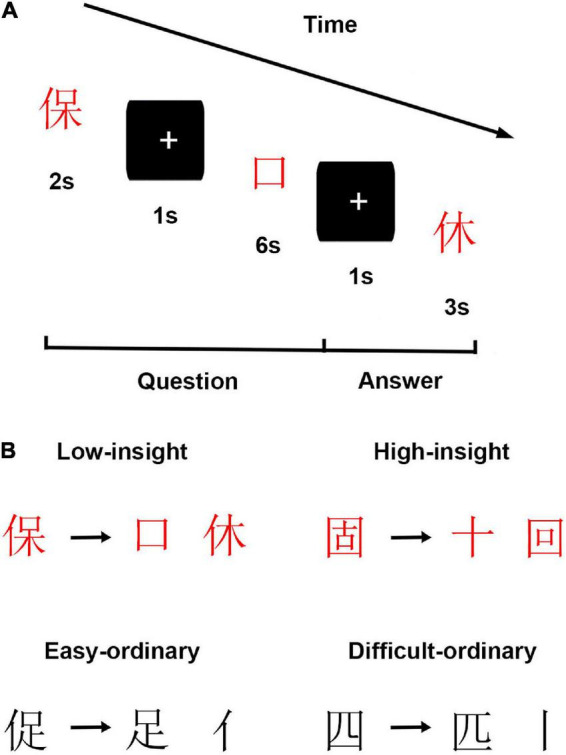
Schematic of experimental design. **(A)** Experimental procedures. **(B)** Exemplars of each condition in insight and filler tasks.

The present study used individuals’ performance on the insightful Chinese character chunk decomposition tasks to measure individuals’ dispositional insight. Figure 1 shows the insight task and materials in the study. Notably, the difficulty of the experimental materials might potentially affect the performance of subjects. For instance, if all the testing materials were too easy or difficult for each subject, the result would reveal ceiling or floor effects. Thus, we created two kinds of insightful Chinese character chunk decomposition which varies in difficulty, including low and high insightful Chinese character chunk decomposition conditions. Specifically, the low insight condition required subjects to transform the given character (e.g., “保”) into the target character (e.g., “口”) by discarding the to-be-removed character (e.g., “休”). Notably, each given character (e.g., “保”) in this condition is a left-right structure, which comprises a radical (e.g., “亻”) and a sub-character (e.g., “呆”). Meanwhile, each sub-character is a top-bottom structure that comprises a radical (e.g., “口”) and a character (e.g., “木”). In the macroscopic view, it is easy for subjects to decompose the radical (e.g., “亻”) from the given character (e.g., “保”) by intuition. In other words, it is easy to separate the radical (e.g., “亻”) and the sub-character (e.g., “呆”), which fulfills the expectations of subjects. Previous studies have verified that this radical-level character decomposition is easy and can be done through ordinary thinking (Luo et al., 2006; Huang et al., 2015). However, in this insight condition, subjects were required to decompose the to-be-removed character (e.g., “休”), not the radical (e.g., “亻”), from the given character (e.g., “保”) to constitute the target character (e.g., “口”). This process violates subjects’ expectations and is creative for subjects to perform. In the high insight condition, subjects were also asked to change the given character (e.g., “固”) into the target character (e.g., “十”) by discarding the to-be-removed character (e.g., “回”). However, in this insight condition, each target character (e.g., “十”) is visually embedded in the to-be-removed character (e.g., “回”). Subjects are unfamiliar with these materials. Thus, they may adopt more creative ways to perform this decomposition.

Filler tasks (i.e., ordinary Chinese character chunk decompositions), also comprising easy and difficult conditions, were included in the present study (Figure 1). In the easy ordinary condition, subjects were asked to perform radical-level Chinese character chunk decompositions, such as decomposing given characters (e.g., “促”) into target characters (e.g., “足”) by discarding the to-be-removed parts (e.g., “亻”). In the difficult ordinary condition, subjects were asked to perform stroke-level Chinese character chunk decompositions, such as decomposing given characters (e.g., “四”) into target characters (e.g., “匹”) by discarding the to-be-removed parts (e.g., “丨”). Previous studies have proved that stroke-level Chinese character chunk decompositions were more complicated than the radical-level ones (Luo et al., 2006; Wu et al., 2013). Notably, we employed the filler tasks to prevent subjects from deliberately using strategies in performing the insight tasks. Furthermore, the filler tasks were randomly intermixed with the insight tasks to vary the charges and keep subjects’ attention on the tasks. In sum, there were four conditions in this study.

The current experiment contained 160 trials, equally divided into two blocks, comprising 20 trials per condition. The sequence of trials was randomized. For each trial, subjects viewed a given character, a target character, and a to-be-removed portion. Firstly, the given character was presented centrally on the screen for 2 s. Then, a fixation cross was presented for 1 s. After that, the target character was presented centrally on the screen for 6 s until participants responded. During this time, subjects were informed about changing the given character into the target character and finding a solution (i.e., finding the to-be-removed portion) as quickly as they could within 6 s by pressing a key. Here, we recorded the subjects’ response times (RTs). Finally, following a fixation cross for 1s, a new portion was shown on the screen. Subjects determined whether this new portion was the same as one had previously found, then pressed the “F” or “J” key as quickly as they could in 3 s. Here, we recorded the subjects’ correct response rates (CRs).

In order to validate whether the insight task can induce an insightful experience or not, additional 20 subjects rated the creativity and “Aha!” experience of the insight and filler tasks on a 5-point scale. Results showed that the insight task (*M* = 3.13, *SD* = 0.37) was significantly higher in creativity than the filler task (*M* = 1.53, *SD* = 0.42, *p* < 0.001). Within the insight task, high insight trials (*M* = 3.44, *SD* = 0.37) were more creative than the low insight trials (*M* = 2.82, *SD* = 0.45, *p* < 0.001). In addition, the insight task (*M* = 3.02, *SD* = 0.38) induced more “Aha!” experience than the filler task (*M* = 1.44, *SD* = 0.29, *p* < 0.001). Within the insight task, high insight trials (*M* = 3.33, *SD* = 0.41) induced more “Aha!” experience than the low insight trials (*M* = 2.70, *SD* = 0.43, *p* < 0.001). Therefore, the insightful Chinese character chunk decomposition was an insight task.

After this behavioral experiment, all subjects received an MRI scan, including anatomical imaging (5 min) and resting-state imaging (8 min). During the resting-state imaging, all subjects were required to relax and stay awake, with their eyes closed.

### Measures

#### Dispositional Insight

For each subject, we took both high and low insight tasks together to estimate the extent of dispositional insight. The mean RTs of both low and high insight tasks were calculated, then the mean CRs of low and high insight tasks were acquired. In the end, an inverse efficiency score (IES: the ratio of mean RTs over mean CRs) was computed and finally log-transformed to an approximately normal distribution (Townsend and Ashby, 1978; Bruyer and Brysbaert, 2011; Reyes and Sackur, 2014). Notably, the lower the transformed IES is, the higher the dispositional insight becomes.

### Personality

In the current study, the Revised NEO Personality Inventory (NEO-PI-R), IPIP-NEO-120, was employed to estimate the personality of each subject (Costa, and McCrae, 1992). Previous studies verified that this NEO-PI-R had high reliability and validity in Chinese populations (Yang et al., 1999; Kong et al., 2015a; Xiang et al., 2016; Kajonius and Mac Giolla, 2017). In brief, the NEO-PI-R measures five different dimensions of personality: neuroticism, extraversion, openness to experience, agreeableness, and conscientiousness. The NEO-PI-R comprises 120 items, with 24 items per dimension. Subjects should respond to each item on a 5-point Likert scale, which ranges from strong disagreement to strong agreement. Notably, negatively worded items were reversely coded before all analyses.

### Image Acquisition

Scanning was conducted on a 3 T Siemens Trio Tim MR scanner with a 12-channel phased-array head coil in SCNU, Guangzhou, China. Briefly, the T1-weighted images were acquired with a magnetization-prepared rapid acquisition gradient-echo (MPRAGE) sequence (TR/TE/FA/thickness: 1900 ms/2.52 ms/9°/1.0 mm; matrix size: 256 × 256; FOV: 256 × 256 mm^2^; 176 sagittal slices). The resting-state fMRI data were obtained using an Echo Planar Imaging (EPI) sequence (TR/TE/FA/thickness: 2000 ms/30 ms/90°/3.5 mm; matrix size: 64 × 64; FOV: 204 × 204 mm^2^; 33 axial slices).

### Voxel-Based Morphometry

The computational anatomy toolbox 12 (CAT 12 toolbox^1^) was adopted to perform the VBM analysis of structural images. In the present study, we utilized the suggested default settings of the CAT 12 toolbox to preprocess the structural images. Detailed descriptions can be found in the manual of the CAT 12 toolbox.^2^ Brie?y, the preprocessing procedure comprised the following steps: (1) correcting for magnetic field inhomogeneities, (2) segmenting the brain into gray matter (GM), white matter (WM), and cerebrospinal fluid (CSF), (3) normalizing the images using the DARTEL algorithm, (4) modulating the images using the Jacobian determinants, (5) smoothing the images with an 8 mm Gaussian kernel (Besteher et al., 2017; Farokhian et al., 2017; Takeuchi et al., 2018). In addition, the total intracranial volume (TIV) was computed in the present study.

### Resting-State Functional Connectivity

The functional brain data were entered into the DPABI software package^3^ and received preprocessing (Yan et al., 2016). Brie?y, the suggested standard procedure incorporated the following steps: (1) removing the first ten images, (2) slice-time correction, (3) head-motion correction, (4) normalizing the images into the MNI template by using the T1 image for coregistration, (5) smoothing the images by using a 4 mm full-width half-maximum (FWHM) Gaussian filter, (6) signal linear detrending, (7) removing nuisance signal (WM and CSF signal, and Friston 24-parameters of head motion) (Yan et al., 2013; Wu et al., 2019), (8) temporal filtering (0.01-0.08 HZ). Notably, this study estimated the 6-parameters of head motion, and all the subjects met the criteria (translation < 2.5 mm and rotation < 2.5°). In addition, the Friston 24-parameters of head motion were also computed, and the mean framewise displacement (FD) of head motion was acquired and subsequently used as a regressor of no interest to be regressed out in the statistical analysis (Yan et al., 2013).

In the RSFC analysis, the brain regions showing significant correlations between the rGMV and dispositional insight were selected as the seed regions to compute the voxel-wise functional connectivity. Firstly, for each seed region, the mean time course of the seed region was calculated. Secondly, we calculated Pearson’s correlation coefficients between the mean time course of the seed region and the time course of all other brain voxels. Thirdly, for normality purposes, the correlation coefficients were transformed to z-scores (i.e., Fisher’s r-to-z transformation). Finally, the seed-based z-RSFC map was acquired for each subject and subsequently entered into statistical analyses.

### Statistical Analyses

#### Voxel-Based Morphometry

In the group-level analysis, a multiple regression model was utilized to explore the structural mechanisms of dispositional insight. In other words, based on the preprocessed structural images, we planned to identify the brain regions in which the rGMV was correlated with dispositional insight (measured by IES). In this model, the IES was used as the regressor of interest. Meanwhile, since several studies indicated that factors of sex, age, and TIV could affect the brain structure, the influence factors of sex, age, and TIV were regressed out (Malone et al., 2015; Lin et al., 2017). For multiple comparisons correction, the statistical maps were thresholded at voxel-wise uncorrected *p* < 0.005 and cluster-extent FWE corrected *p* < 0.05 (Hayasaka et al., 2004; Wang et al., 2018).

#### Resting-State Functional Connectivity

In the group-level analysis, a multiple regression model based on the z-RSFC maps was built. We planned to find out the brain regions showing correlations between the RSFC and dispositional insight (measured by IES). Specifically, in this model, the IES was entered as the regressor of interest; gender, age, and mean FD of head motion were entered as the regressors of no interest (Wu et al., 2019). The multiple comparisons correction here was the same as the one used in the VBM analysis.

### Mediation Analysis

To examine whether the personality can mediate the linkage between the dispositional insight and brain structure or not, a mediation model was built by using the PROCESS macro (Preacher and Hayes, 2008; Xiang et al., 2016). In this model, the brain structure (i.e., rGMV value) was used as an independent variable (X), and the dispositional insight (i.e., IES value) was used as a dependent variable (Y). Then, the personality was set as a mediating variable (M). We aimed to test the effects of X on Y through M. Following previous studies (Li et al., 2014; Xiang et al., 2016), we didn’t control other personality dimensions when entering one personality dimension as M. Notably, we utilized a bootstrap procedure (*n* = 10000) to validate the impact of X on Y through M. A 95% confidence interval which does not incorporate 0 implies that the indirect effect is significant at the 0.05 level.

## Results

### Behavioral Results

Firstly, we found that the high insight condition was longer in RTs [*t* (43) = 5.93, *p* < 0.001] and lower in CRs [*t* (43) = -5.56, *p* < 0.001] than the low insight condition, reflecting the validity of our manipulations on the degree of insight. Notably, we used RTs and CRs to calculate the IES (an index of dispositional insight). Secondly, the mean (SD), range, skewness, and kurtosis of the IES and the score of each personality were computed and listed in Table 1. The kurtosis and skewness of each score varied from -1 to +1, suggesting normality of the score (Marcoulides and Hershberger, 1997; Xiang et al., 2016).

**TABLE 1:** Descriptive statistics of dispositional insight (estimated by IES) and each personality.

	Means (SD)	Range	Skewness	Kurtosis	Correlation with IES
IES	3.18 (0.16)	2.91–3.54	−0.02	−0.81	N/A
Neuroticism	73.25 (10.17)	49.00–98.00	0.48	0.49	−0.47**
Extraversion	77.39 (6.53)	65.00–96.00	0.09	0.36	−0.01
Openness to experience	82.39 (7.42)	62.00–97.00	-0.17	0.52	−0.29
Agreeableness	84.91 (9.52)	58.00–107.00	−0.16	0.61	0.04
Conscientiousness	78.23 (8.18)	62.00–90.00	−0.29	−0.98	0.08

“**” represents p < 0.01. N/A, not applicable; SD, standard deviation; IES, inverse efficiency score.

### Voxel-Based Morphometry

**FIGURE 2:**
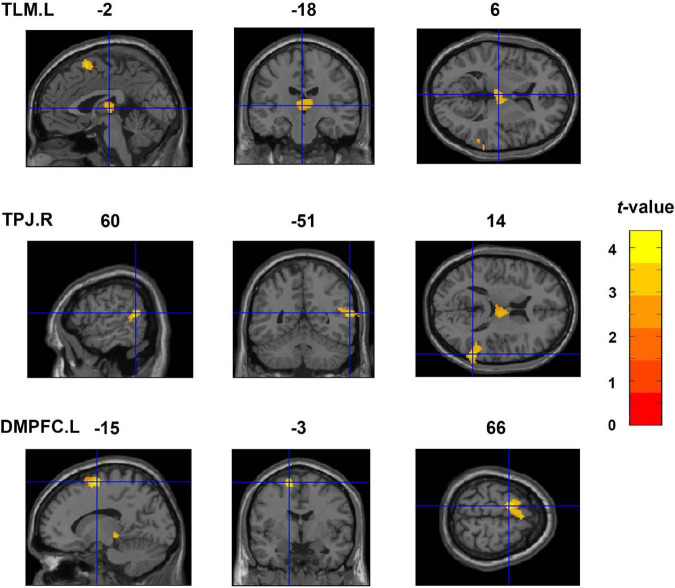
Whole-brain VBM analyses showing three clusters exhibiting significant and positive correlations with the IES (an index of dispositional insight). These clusters include the TLM.L, TPJ.R, and DMPFC.L. Coordinates are shown in the MNI space. TLM.L, left thalamus; TPJ.R, right temporoparietal junction; DMPFC.L, left dorsal medial prefrontal cortex; VBM, voxel-based morphometry; IES, inverse efficiency score; MNI, Montreal Neurological Institute.

Figure 2 shows the findings of the whole brain VBM analysis. We found that the IES was significantly and positively correlated with rGMV in the left thalamus (TLM.L), TPJ.R, and left dorsal medial prefrontal cortex (DMPFC.L). Details of these clusters are listed in Table 2.

**TABLE 2:** Clusters exhibiting significant and positive correlations with the IES in the VBM analyses.

Regions	Cluster size	MNI coordinates			*t* value
	(voxels)	x	y	z	
TLM.L	1063	-2	-18	6	3.44
TPJ.R	1287	60	-51	14	4.23
DMPFC.L	1203	-15	-3	66	4.38

*The t value denotes the statistical difference in a cluster. TLM.L, left thalamus; TPJ.R, right temporoparietal junction; DMPFC.L, left dorsal medial prefrontal cortex; VBM, voxel-based morphometry; IES, inverse efficiency score; MNI, Montreal Neurological Institute.*

### Resting-State Functional Connectivity

**FIGURE 3:**
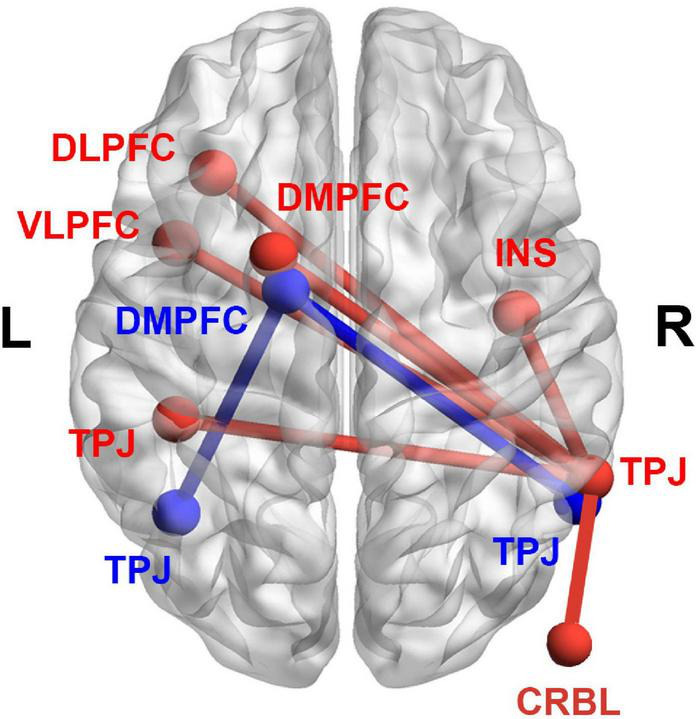
Results derived from the seed-based RSFC analysis. Significant correlations between the RSFC and the IES (an index of dispositional insight). Red color denotes the RSFC seeded from TPJ.R. The RSFC of TPJ.R-TPJ.L, TPJ.R-DLPFC.L, TPJ.R-VLPFC.L, TPJ.R-DMPFC.L, TPJ.R-INS.R, and TPJ.R-CRBL.R were significantly and positively correlated with IES. Blue color denotes the RSFC seeded from DMPFC.L. The RSFC of DMPFC.L-TPJ.R, and DMPFC.L-TPJ.L were significantly and positively correlated with IES. RSFC, resting-state functional connectivity; IES, inverse efficiency score; TPJ.R, right temporoparietal junction; DMPFC.L, left dorsal medial prefrontal cortex; DLPFC.L, left dorsolateral prefrontal cortex; VLPFC.L, left ventrolateral prefrontal cortex; INS.R, right insula; CRBL.R, right cerebellum.

Figure 3 shows the results of the seed-based RSFC analysis. The brain regions of TLM.L, DMPFC.L, and TPJ.R derived from VBM analysis were selected as seed regions. Results showed that the RSFC between the seed region of DMPFC.L and the regions of TPJ.L and TPJ.R was significantly and positively correlated with the IES. Moreover, the RSFC between the seed region of TPJ.R and the regions of the left dorsolateral prefrontal cortex (DLPFC.L), left ventrolateral prefrontal cortex (VLPFC.L), DMPFC.L, TPJ.L, right insula (INS.R), and right cerebellum (CRBL.R) was also significantly and positively correlated with the IES. Details of these results can be found in Table 3.

**TABLE 3:** Significant and positive correlations between the RSFC and IES (an index of dispositional insight).

Seed regions	RSFC regions	Cluster size	MNI coordinates			*t* value
			
		(voxels)	x	y	z	
TPJ.R	TPJ.L	198	-42	-36	42	4.27
	DLPFC.L	148	-33	27	15	4.73
	VLPFC.L	146	-42	9	15	4.18
	DMPFC.L	263	-18	6	66	4.36
	INS.R	121	42	-9	24	4.71
	CRBL.R	89	15	-75	-45	4.79
DMPFC.L	TPJ.L	152	-42	-60	21	3.87
	TPJ.R	93	57	-57	12	3.91

*In the calculations, we selected the three clusters listed in Table 2 as the seed regions, and calculated their RSFC in the whole brain to obtain the regions with significant correlations between the RSFC and IES. The t value denotes the statistical difference in a cluster. RSFC, resting-state functional connectivity; IES, inverse efficiency score; TPJ.R, right temporoparietal junction; DMPFC.L, left dorsal medial prefrontal cortex; DLPFC.L, left dorsolateral prefrontal cortex; VLPFC.L, left ventrolateral prefrontal cortex; INS.R, right insula; CRBL.R, right cerebellum; MNI, Montreal Neurological Institute.*

### Mediation Analysis

**FIGURE 4:**
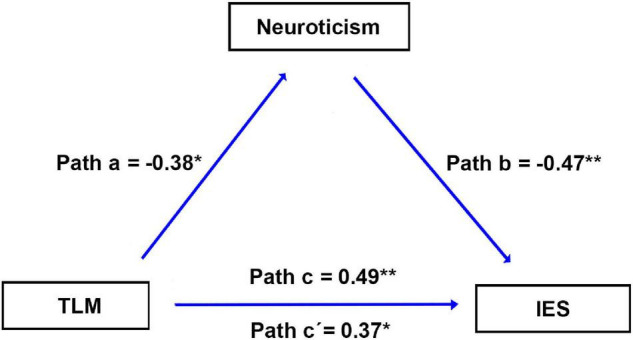
Results of the mediation analysis. Neuroticism mediates the association between the brain structure and IES. Paths a, b, c, and c’ denote standard regression coefficients. Path a: rGMV in the TLM.L is significantly correlated with neuroticism. Path b: neuroticism is significantly correlated with IES. Path c: rGMV in the TLM.L is significantly correlated with IES. Path c’: after regressing out neuroticism, rGMV in the TLM.L is significantly correlated with IES. IES, inverse efficiency score; rGMV, regional gray matter volume; TLM.L, left thalamus.

Figure 4 shows that the personality of neuroticism mediates the relationship between the cluster of TLM.L and dispositional insight (estimated by IES). Specifically, we first investigated the relationship between each personality (i.e., neuroticism, extraversion, openness to experience, agreeableness, or conscientiousness) and dispositional insight. We found that only neuroticism exhibited a significant correlation with IES (*r* = -0.47, *p* = 0.001, Table 1). Subsequently, we tested whether brain structures associated with dispositional insight (TLM.L, DMPFC.L, and TPJ.R) could be correlated with neuroticism. We detected that only the rGMV in the TLM.L showed a significant correlation with neuroticism (*r* = -0.38, *p* = 0.012, see Figure 4). The findings implied that neuroticism was associated with both dispositional insight and rGMV in the TLM.L. Finally, to clarify this relationship further, we conducted a mediation model to test whether neuroticism can mediate the linkage between the rGMV in the TLM.L and dispositional insight or not. Specifically, we examined the linkage between the rGMV in the TLM.L and the IES without regressing out neuroticism. We found that rGMV in the TLM.L exhibited a significant correlation with IES (β = 0.49, *p* = 0.001). When regressing out the neuroticism, the correlation between the TLM.L and IES was still significant but decreased a little (β = 0.37, *p* = 0.011). In addition, the bootstrap procedure (*n* = 10000) validated that neuroticism mediated the linkage between the TLM.L volume and IES (95% confidence interval = [0.049, 1.406], *p* < 0.05).

## Discussion

The present study aimed to study the changed brain structure and RSFC associations with dispositional insight. The VBM analysis found that IES (an index of dispositional insight) was positively correlated with the rGMV in the TLM.L, TPJ.R, and DMPFC.L. In the RSFC analysis, we found that IES was positively correlated with the RSFC of TPJ.R-TPJ.L, TPJ.R-DLPFC.L, TPJ.R-VLPFC.L, TPJ.R-DMPFC.L, TPJ.R-INS.R, TPJ.R-CRBL.R, DMPFC.L-TPJ.L, and DMPFC.L-TPJ.R. The RSFC analysis was to complement the findings of VBM analysis. Notably, the lower the IES is, the higher the dispositional insight becomes. Thus, dispositional insight is negatively correlated with the rGMV and RSFC of relevant brain regions. Finally, the mediation analysis showed that neuroticism mediated the linkage between the TLM.L volume and IES.

The TLM is a hub region widely connected with the whole brain (Van Den Heuvel and Sporns, 2011). It is mainly responsible for relaying information and involves reward processing (Gazzaniga, 2004; Boccia et al., 2015; Jung et al., 2015; Takeuchi et al., 2017; Cristofori et al., 2018; Tik et al., 2018). Previous studies on divergent thinking found that reduced dopamine receptors in the TLM were associated with reduced information filtering and reduced inhibition of prefrontal neurons, which contributed to performances requiring increased flexibility (Jung et al., 2015). A previous study indicated that expected reward can facilitate insight problem solving (Cristofori et al., 2018). In insight problem solving, problem solvers needed to perform a variety of solution attempts to find the correct solution (Bowden and Jung-Beeman, 2003). Therefore, the dispositional insight correlated with TLM may indicate a relationship between dispositional insight and information searching, and reward processing. In addition, TPJ is generally believed to act as a core region involved in attention reorienting and forming novel associations (Qiu et al., 2008; Berkowitz and Ansari, 2010; Chang et al., 2013). Previous studies indicated that attentional resources should be deployed to prevent reorienting to distracting stimuli during divergent thinking (Corbetta et al., 2008; Benedek et al., 2014b). Task-fMRI studies suggested that TPJ prevented irrelevant objects from being attended and formed new associations in the insight process (Qiu et al., 2008; Tang et al., 2015). The dispositional insight correlated with TPJ may indicate a linkage between dispositional insight and attention reorienting, and forming associations. Importantly, DMPFC plays a critical role in conflict regulation and cognitive control (Egner and Hirsch, 2005; Krebs et al., 2013; Benedek et al., 2014a). Our findings were in line with previous studies on creativity (Beaty and Silvia, 2012; Benedek et al., 2013, 2014a). For example, generating creative ideas was associated with top-down control and executive processes (Benedek et al., 2013). In creative thinking, individual need to inhibit numerous competing responses before a suitable response was found (Benedek et al., 2014a). Moreover, inhibiting the dominant mental sets and forming new representations were crucial for episode insight (Bowden and Jung-Beeman, 2003). Taken together, altered brain structure in the DMPFC might be associated with dispositional insight.

In addition, dispositional insight was negatively correlated with the RSFC of TPJ.R-TPJ.L, TPJ.R-DLPFC.L, TPJ.R-VLPFC.L, TPJ.R-DMPFC.L, TPJ.R-INS.R, TPJ.R-CRBL.R, DMPFC.L-TPJ.L, and DMPFC.L-TPJ.R. Lateral prefrontal cortex, including the DLPFC and VLPFC, involves inhibition of dominant but irrelevant responses and cognitive flexibility (Derrfuss et al., 2005; Volle et al., 2011). Usually, the cerebellum is linked to working memory and executive functions (Ravizza et al., 2005; Keren-Happuch et al., 2014). Previous studies noted that cognitive flexibility made it possible for problem solvers to think away from ordinary thinking, which was in line with our present study (Munakata et al., 2011; Gonen-Yaacovi et al., 2013). Insula is associated with subjective emotion (Quartz, 2009; Singer et al., 2009). In the insightful tasks, insight solutions often evoked “aha” feelings (Bowden and Jung-Beeman, 2007; Gonen-Yaacovi et al., 2013). Thus, we speculate that activations in the lateral prefrontal cortex, cerebellum, and insula may imply a link between the dispositional insight, cognitive inhibition, and “aha” feelings.

Notably, previous studies indicated that the involvement of the frontal and parieto-temporal networks might be associated with creative generation tasks (Takeuchi et al., 2010; Gonen-Yaacovi et al., 2013; Lin et al., 2020). In a task-fMRI study, an insight solution involved more robust functional connectivity between the prefrontal cortex and temporal cortex than a non-insight solution (Zhao et al., 2014). Consistent with these studies, we found that the RSFC between the frontal and parieto-temporal regions involved dispositional insight. In sum, we infer that specific brain structures associated with RSFC contribute to dispositional insight.

Finally, we detected that neuroticism mediated the link between the TLM.L and dispositional insight. Specifically, our findings highlighted that rGMV of the TLM.L was significantly correlated with neuroticism. Several studies have revealed associations between the TLM and neuroticism (Takano et al., 2007; Servaas et al., 2013; Kong et al., 2015b). For example, there was a relationship between serotonin transporter binding in the TLM and neuroticism (Takano et al., 2007). Moreover, imaging research suggested that neuroticism was related to structural variations in the TLM (Kong et al., 2015b). Previous studies revealed that individuals with higher neuroticism tended to accompany anxiety and worry emotional states (Carver et al., 2000; Leung et al., 2014). The emotional states facilitated analyses of complex problems and attainments of desired outcomes to prevent adverse effects (Andrews and Thomson, 2009; Leung et al., 2014). Besides, researches suggested that individuals with high scores on neuroticism tended to dwell on problems and recruit many rumination-related processes, such as worrying, which were helpful for creative problem-solving (Perkins et al., 2015). Individuals with higher neuroticism could generate many unusual thoughts in the idea generation tasks (Leung et al., 2014). These studies imply that neuroticism might facilitate insightful performances, in line with our findings suggested that neuroticism was positively correlated with dispositional insight. It is rational that neuroticism plays a mediation role in the relationship between TLM and dispositional insight.

Considering the important role of TLM in dispositional insight, the TML was further decoded for cognitive terms with Neurosynth toolbox^4^ to depict the functional validity of the TLM (Rubin et al., 2017). The top 50 terms were first selected, from which we only reported the top five terms related to specific cognitive and emotional functions in descending order. Results showed that terms were closely correlated with anticipation, monetary reward, hypoactivation, sensations, and mood. This finding is in line with previous studies, where insight could involve the processes of reward, active searching, and mood (i.e., “aha” feelings) (Cristofori et al., 2018; Tik et al., 2018). Therefore, the dispositional insight correlated with TLM may indicate a relationship between dispositional insight, reward processing, active searching, and emotional processing. However, the results from the decoding analysis were descriptive, and further studies need to clarify this issue.

The present study found that dispositional insight was negatively correlated with the rGMV and RSFC of specific brain regions. Consistently, evidence from previous studies supported the association (Chen et al., 2014; Li et al., 2014). For instance, Chen et al. (2014) found that trait creativity was negatively correlated with the rGMV in the dorsal ACC and the RSFC between the dorsal ACC and MSFC. However, there were contradictory studies indicating an inconsistent association (Gansler et al., 2011; Takeuchi et al., 2012; Beaty et al., 2014; Li et al., 2014; Zhao et al., 2014). For instance, Li et al. (2014) reported that creative subjects had increased rGMV in the middle temporal gyrus. Takeuchi et al. (2012) showed that trait creativity was positively related to the RSFC between the MPFC and posterior cingulate cortex. In fact, it is hard to explain the relationship between decreased or increased rGMV and creativity. Previous studies indicated that increased rGMV reflects neuroplasticity, which may be helpful for task performance (Draganski et al., 2006; Chen et al., 2014). However, other studies showed that decreased rGMV reflects neuronal pruning processes, which may be related to better cognitive performance (Kanai and Rees, 2011; Duan et al., 2012). Thus, we speculated that neuronal pruning processes might be the reason for decreased rGMV, contributing to high dispositional insight in this study. In addition, previous studies suggested a consistent relationship between gray matter alteration and RSFC (Chen et al., 2014; Niu et al., 2018). For instance, in a trait creativity study, researchers found that decreased rGMV in the dorsal ACC appears to result in the reduced RSFC within the salience network (Chen et al., 2014). Therefore, we deduced that decreased rGMV might be the reason for decreased RSFC, contributing to high dispositional insight in this study. Finally, we didn’t estimate Chinese language abilities of subjects in the present study. However, we recruited the student subjects randomly, who have passed the college entrance examination. We assumed that they have similar language abilities.

## Conclusion

The present study utilized a multi-modal imaging method to reveal the structural and functional mechanisms underlying dispositional insight. We found that rGMV in the TLM, TPJ, and DMPFC was related to dispositional insight. Altered RSFC of the frontal and parieto-temporal regions also involved dispositional insight. In addition, neuroticism was proved to be a mediational mechanism explaining the link between the TLM volume and dispositional insight. Our study provides a new understanding of the brain structure and functions underlying dispositional insight.

## Data Availability Statement

The raw data supporting the conclusions of this article will be made available by the authors, without undue reservation.

## Ethics Statement

The studies involving human participants were reviewed and approved by The Research Ethics Review Board of South China Normal University. The patients/participants provided their written informed consent to participate in this study.

## Author Contributions

JL and LM developed the study concept. QC, MZ, and YC performed testing and data collection. JL and YC performed the data analysis and interpretation. JL, JX, and YC drafted the manuscript. JL, JX, and LM provided critical revisions. All authors approved the final version of the manuscript for submission.

## Conflict of Interest

The authors declare that the research was conducted in the absence of any commercial or financial relationships that could be construed as a potential conflict of interest.

## Publisher’s Note

All claims expressed in this article are solely those of the authors and do not necessarily represent those of their affiliated organizations, or those of the publisher, the editors and the reviewers. Any product that may be evaluated in this article, or claim that may be made by its manufacturer, is not guaranteed or endorsed by the publisher.

## Footnotes

1
http://www.neuro.uni-jena.de/cat/

2
http://www.neuro.uni-jena.de/cat12/CAT12-Manual.pdf

3
http://rfmri.org/dpabi

4
https://www.neurosynth.org/
